# Induction of Prostate Tumours in Mice

**DOI:** 10.1038/bjc.1947.7

**Published:** 1947-03

**Authors:** E. S. Horning, L. Dmochowski

## Abstract

**Images:**


### INDUCTION OF PROSTATE TUMOURS IN MICE.

E. S. HORNING AND L. DMOCHOWSKI.

*From the Laboratories of the Imperial Cancer Research Fund, Mill Hill, N.W. 7.*

Received for publication December 3, 1946.

THE object of this investigation was the induction of prostate tumours in mice by 20-methylcholanthrene.

Pure line strains of mice were used so that transplantation of the experimental tumours might lead, if successful, to the continuous propagation of uniform tumours for subsequent experiments on the effects of oestrogen upon tumour growth.

### THE STRUCTURE OF THE NORMAL PROSTATE GLAND IN MICE.

The nomenclature of the prostate lobes in rodents has led to much controversy (Rauther, 1903; Disselhorst, 1904; Walker, 1910; Burrows, 1934; Korenchevsky and Dennison, 1935).

The glands, generally referred to as the lobes of the prostate in the mouse, have been described by Deanesly and Parkes (1933), as consisting of paired anterior, ventral and dorsal lobes, together with a small median gland—the ampullary lobe. Fekete (1941) described this tubular median gland as lying adjacent to and opening into the ampulla of the ductus deferens.

It is often suggested that the series of glands comprising the prostate of rodents are not necessarily homologous with the human prostate. Nevertheless, these structures in rodents and in man arise by similar outgrowths from the wall of the urethra, and there are clear histological similarities between the components of the rodent and human glands.

Thus we find in the mouse: (1) The anterior prostates which are paired lobes, sometimes referred to as the coagulating glands (Burrows, 1945), but described by Deanesly and Parkes (1933) as the anterior prostates. They are embedded in the peritoneal sheath of the seminal vesicles, and open into the anterior dorsal wall of the urethra by means of two ducts. They are lined by simple columnar epithelium. The mucous membrane is thrown into folds which project into the lumen, their appearance depending upon the functional activity of the gland (Fig. 1).

The anterior prostate secretes an enzyme which coagulates the seminal fluid, hence the alternative name "coagulating gland." This gland is considered by some investigators (Callow and Deanesly, 1935; Moore, 1939) to be part of the true prostate, because it has a similar embryological origin as the ventral and dorsal prostates. It also possesses the same histological structure as the dorsal lobes.

(2) The dorsal prostates are considerably smaller in size than the anterior glands, having several ducts, all of which open into the dorsal wall of the urethra. Their histological structure is similar to that of the anterior lobes, except that the epithelium of the dorsal lobes is relatively free from folds (Fig. 2).

(3) The ventral prostates are lined by a low columnar epithelium (Fig. 3). There are four ducts, all of which enter the ventral wall of the urethra near the bladder.

### METHODS.

These experiments were confined to mice of R III and Strong A strains. The average age of the mice was 6 months. Methylcholanthrene was introduced into the prostate gland in the following manner:

Under general anaesthesia, a median abdominal incision was made, exposing the prostate lobes. Using a dissecting microscope, 0·1 c.c. of a 1·5 per cent solution

---

### DESCRIPTION OF PLATES.

### PLATE I.

FIG. 1.—Anterior prostate or coagulating gland of a 6 months' old Strong A mouse. Masson's light green-eosin.

FIG. 2.—Dorsal prostate of a 6 months' old Strong A mouse. Masson's light green-eosin.

FIG. 3.—Ventral prostate of a 6 months' old Strong A mouse. H. and E.

FIG. 4.—Technique by which 20-methylcholanthrene is injected into the dorsal prostatic lobe.

FIG. 5.—Typical tumour of the prostate gland in a Strong A mouse 4 months after the injection of methylcholanthrene.

FIG. 6.—Action of 20-methylcholanthrene on the epithelium of the anterior prostatic lobe in a Strong A mouse. Cells 3–5 layers deep, retaining their glandular activity, some showing early changes towards squamous epithelium. H. and E.

FIG. 7.—The same at a later stage. Cells acquiring more pronounced squamous metaplasia. H. and E.

FIG. 8.—The same after 3 weeks' time. Development of stratified squamous epithelium, 10 or more layers deep, keratin pearl formation still absent. H. and E.

### PLATE II.

FIG. 9.—The same under higher magnification; cells have broken through the basement membrane. H. and E.

FIG. 10.—The same at a later stage; keratin pearl formation has taken place. H. and E.

FIG. 11.—Action of stilboestrol pellets (15 mg.) on the epithelium of the anterior prostatic lobe of a Strong A mouse. After 3 weeks' time, squamous metaplasia of glandular epithelium and formation of keratin pearls. H. and E.

FIG. 12.—Squamous cell carcinoma with keratin formation of a prostate in a Strong A mouse after 5 months' treatment with methylcholanthrene. H. and E.

FIG. 13.—Similar type of tumour with alveolar structure partially preserved, three months' treatment with methylcholanthrene. H. and E.

FIG. 14.—A diffuse carcinoma in a Strong A mouse after 5 months' treatment with methylcholanthrene. H. and E.

FIG. 15.—Carcinoma with alveolar structure preserved and metaplastic cells; 4½ months' treatment with methylcholanthrene. H. and E.

FIG. 16.—Sarcoma of the prostate in an R III mouse after 6 months' treatment with methylcholanthrene.

Horning and Dmochowski.

1

2

3

4

5

6

7

8

Horning and Dmochowski.

of 20-methylcholanthrene in warm lard, was injected into either the dorsal or anterior prostates. Care was taken not to remove the needle of the syringe until the lard had set, in order to confine the carcinogens to the interior of the glandular tissues (Fig. 4).

The tumours were usually detected by palpation, or sometimes by the signs of distention of the bladder and retention of urine. Death from bilateral hydronephrosis occurred, however, in some mice before transplantation could be undertaken. The tumours which were transplanted grew successfully for many generations.

Primary and transplantable tumours were fixed in alcoholic Bouin, embedded in paraffin, and stained with haematoxylin and eosin, or by Masson's light green eosin technique.

### RESULTS.

No detailed description of the microscopic appearance of the tumours need be given. They varied between ½ to ¾ of an inch in diameter and frequently more than a single prostate lobe was involved.

A tumour of average size is shown in Fig. 5, in which it can be seen as a large central mass affecting all the prostatic lobes.

The number of mice used in the experiments and the incidence and types of tumours are shown in Table I.

TABLE I.

| Strain of mice. | Number of mice. | Number of tumours. | Number of carcinomata. | Average time of appearance (months). | Number of sarcomata. | Average time of appearance (months). |
|---|---|---|---|---|---|---|
| Strong A | 46 (8)* | 22 | 10 | 4·0 | 12 | 4·8 |
| R III | 46 (14)* | 17 | .. | .. | 17 | 7·0 |

* Numbers in brackets indicate the number of mice which died before the earliest tumour appeared.

As can be seen from Table I, both carcinomata and sarcomata of the prostate gland were induced in mice of Strong A strain, whilst in mice of R III strain, only sarcomata developed.

### HISTOGENESIS.

*Carcinoma.*

Examination of the prostate following treatment with 20-methylcholanthrene showed the columnar epithelium undergoing squamous metaplasia. This change took place fairly rapidly in mice of the Strong A strain, leading to an epithelium 3–5 cells in depth. Some cells still retained their glandular activity (Fig. 6). Later phases of this metaplasia are shown in Fig. 7 and 8.

After 3 weeks the epithelium formed layers 5–10 cells thick with some of the basal cells infiltrating individually or in groups into the adjacent stroma (Fig. 9). At a still later stage keratin pearl formation occurred (Fig. 10).

It should be noted that the series of changes in the prostatic epithelium, following a single injection of methylcholanthrene are almost identical with those produced by prolonged treatment with stilboestrol in Strong A mice (Horning, 1946) (Fig. 11).

With oestrogen administration, however, the metaplasia and keratin pearl formation is never followed by malignant changes which take place after treatment with 20-methylcholanthrene.

Fig. 13, 14 and 15 show the types of tumours induced.

### DISCUSSION.

Moore and Melchionna (1937*a*, 1937*b*) induced tumours of the prostate gland in rats of unknown genetic constitution, by injecting 3:4-benzypyrene into the anterior prostate lobe. Recently Dunning, Curtis and Segaloff (1946) by implanting pellets of compressed methylcholanthrene crystals into the prostate gland of rats, induced squamous-celled carcinomas. One of these squamous tumours was found to be transplantable and regularly to form metastases in the lungs, lymph nodes and skeleton. The epithelial changes which followed were similar to those found in the present experiments with Strong A mice.

While in the rat the anterior lobes differ histologically (and also functionally) from the rest of the prostate (Moore, Price and Gallagher, 1930), in the mouse the anterior lobes are similar in structure to the dorsal lobes. In the present experiments, therefore, 20-methylcholanthrene was injected either into anterior or dorsal lobes, with similar results.

Moore and Melchionna (1937*a*, *b*) observed that sarcomata were induced in rat prostate glands in which carcinomata also developed ; similar results were not obtained in these experiments.

There was a similar average time of appearance for both carcinomata and sarcomata in Strong A mice. In the R III strain, however, the tumours (sarcomata) developed at a later average age. The earliest tumours appeared after three months following the injection of methylcholanthrene in both strains of mice. Metastases were not observed, but the invasive growth into the surrounding tissues, and progressive growth after transplantation, leave no doubt that the tumours induced by 20-methylcholanthrene, were malignant.

In attempting to compare the tumours experimentally induced by carcinogens in mice and rats, with those arising spontaneously in man, it should be noted that the common form of carcinoma in man is an adeno-carcinoma. Carcinoma simplex is occasionally found, while sarcomata are rare in adult man, although they have been recorded in children (Fowler, 1942).

In rodents, spontaneous cancer of the prostate so far as we are aware is unknown ; although some experimental tumours in mice and rats retained a glandular structure, there was generally a tendency to metaplasia and keratinization rather than a growth of ordinary adeno-carcinoma.

### SUMMARY.

(1) Transplantable tumours of the prostate gland were induced in Strong A and R III mice following a single injection of 20-methylcholanthrene in lard into the anterior and dorsal lobe.

(2) Squamous metaplasia of the glandular epithelium occurred in both strains, but was followed by malignant proliferation of the metaplastic cells, only in the Strong A strain.

(3) In Strong A mice both carcinoma and sarcomata were found, whereas in R III mice only sarcomata developed.

(4) Carcinomata were of the squamous-cell type ; sarcomata, in both strains, were of the spindle and polymorphic cell type.

### REFERENCES.

Burrows, H.—(1934) *Nature*, **134**, 751.—(1945) 'Biological Actions of Sex Hormones,' Cambridge (University Press), p. 215.
Callow, R. K., and Deansely, R.—(1935) *Biochem. J.*, **29**, 1424.
Deanesly, R., and Parkes, A. S.—(1933) *J. Physiol.*, **78**, 442.
Disselhorst, R.—(1904) *Oppel. Lehrbuch. Vergl. Mikr. Anat. Wirbelt.*, **4**, 309.
Dunning, W. F., Curtis, M. R., and Segaloff, A.—(1946) *Cancer Res.*, **6**, No. 5, 256.
Fekete, E.—(1941) 'Biology of Laboratory Mouse,' edited by G. D. Snell. Philadelphia (Blakiston Press), p. 134.
Fowler, H. A.—(1942) *J. Urol.*, **47**, 16.
Horning, E. S.—(1947) *Quart. J. micr. Sci.*, **88**, 2.
Korenchevsky, V., and Dennison, M.—(1935) *Biochem. J.*, **20**, 1720.
Moore, C. R., Price, D., and Gallagher, I. F.—(1930) *Amer. J. Anat.*, **65**, 109.
*Idem.*—(1939) 'Sex and Internal Secretions,' edited by E. Allen. 2nd ed. London (Baillière, Tindall & Cox), p. 402.
Moore, R. A., and Melchionna, R. H.—(1937*a*) *Amer. J. Cancer*, **30**, 731.—(1937*b*) *Amer. J. Path.*, **13**, 659.
Rauther, M.—(1903) *Jena Zeit. Naturwiss.*, **31**, 377.
Walker, G.—(1910) *Bull. Johns Hopk. Hosp.*, **21**, 182.

---


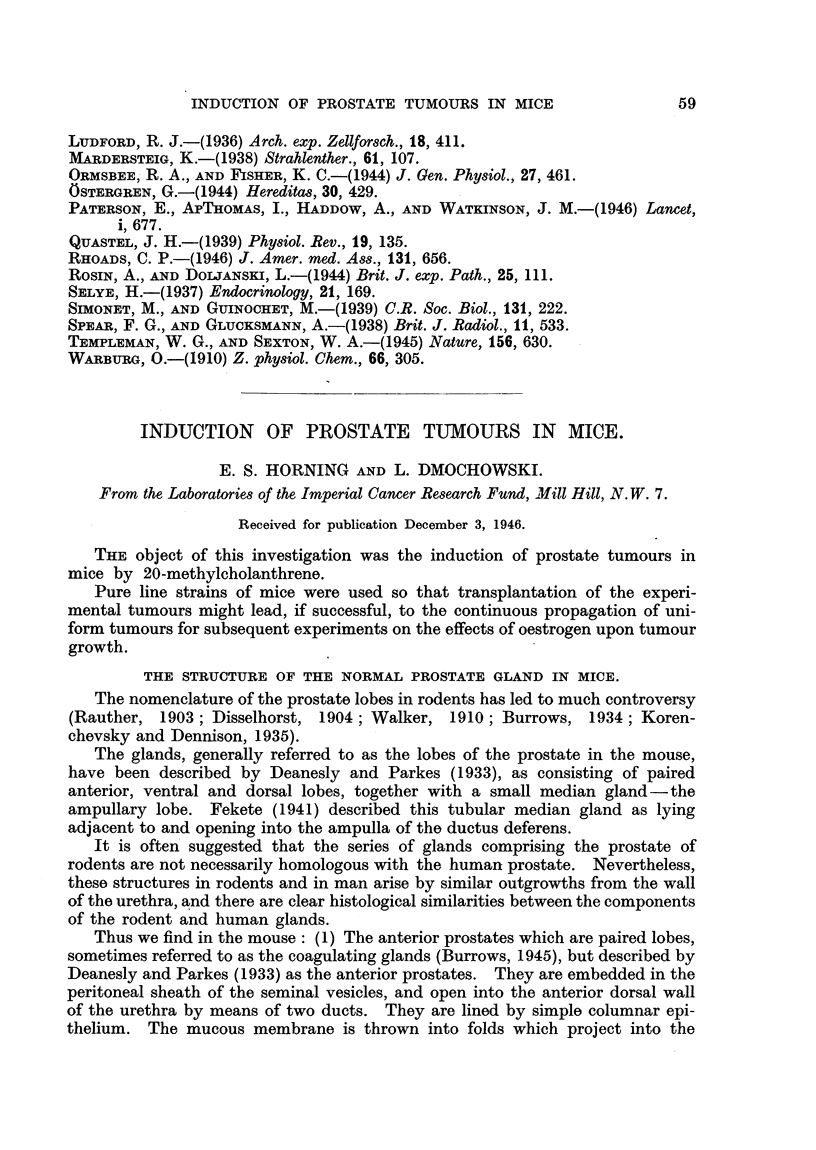

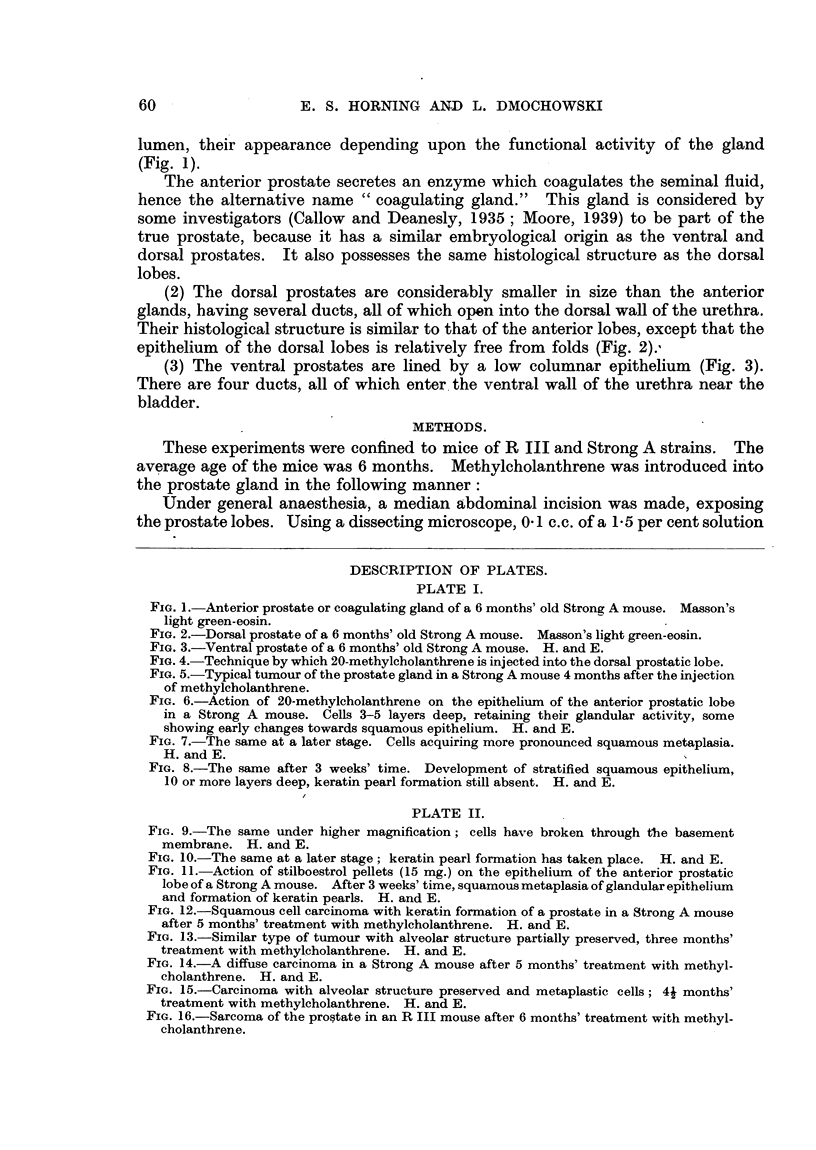

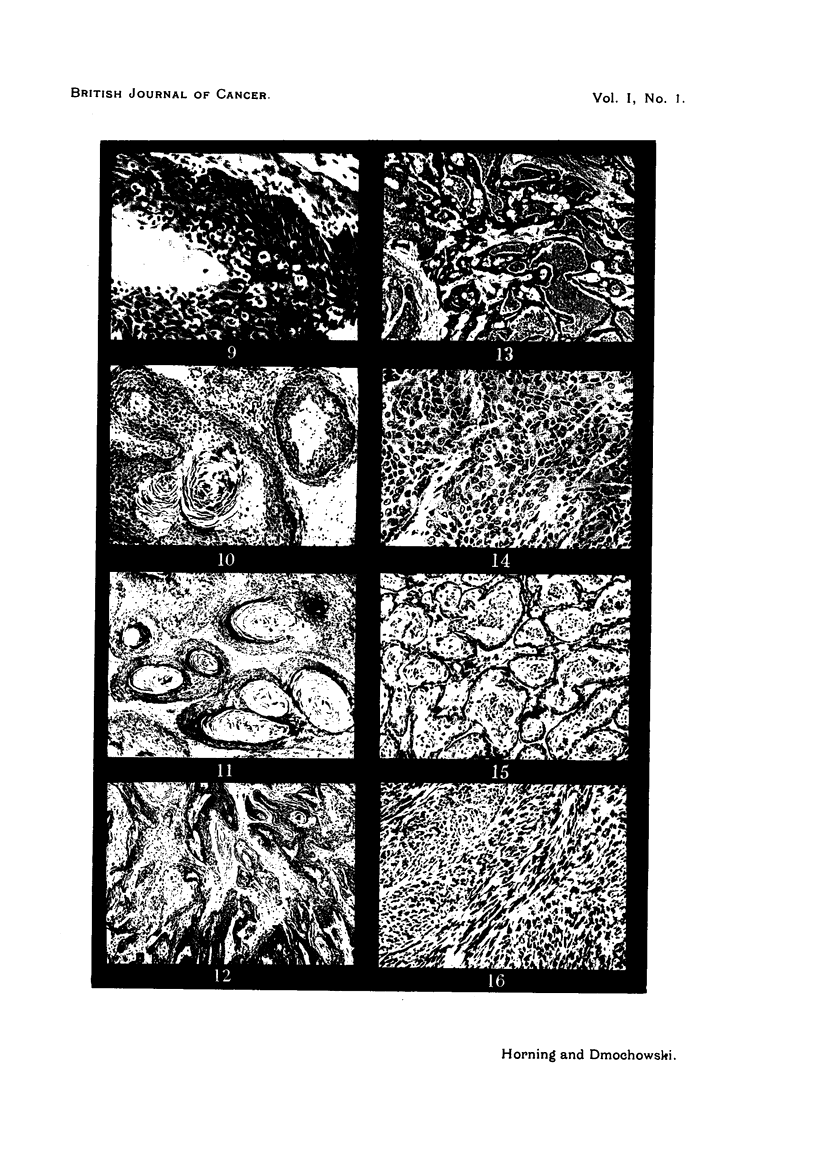

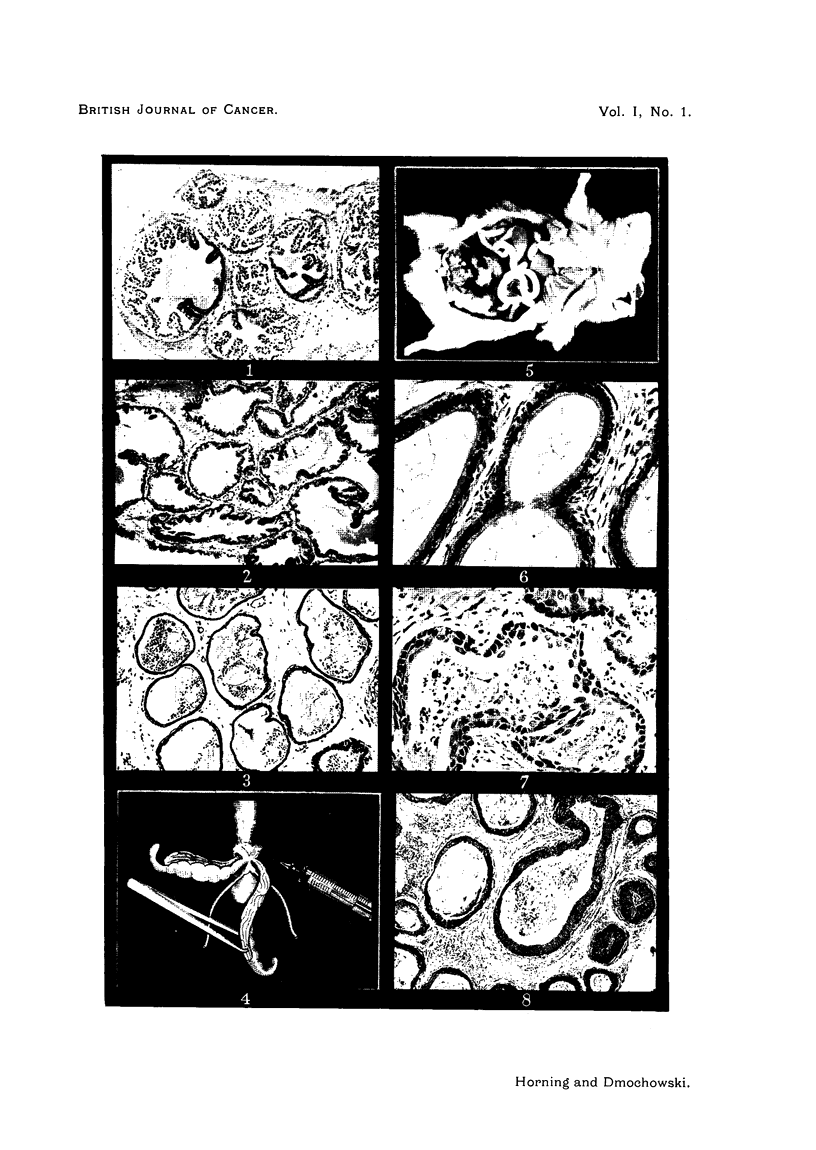

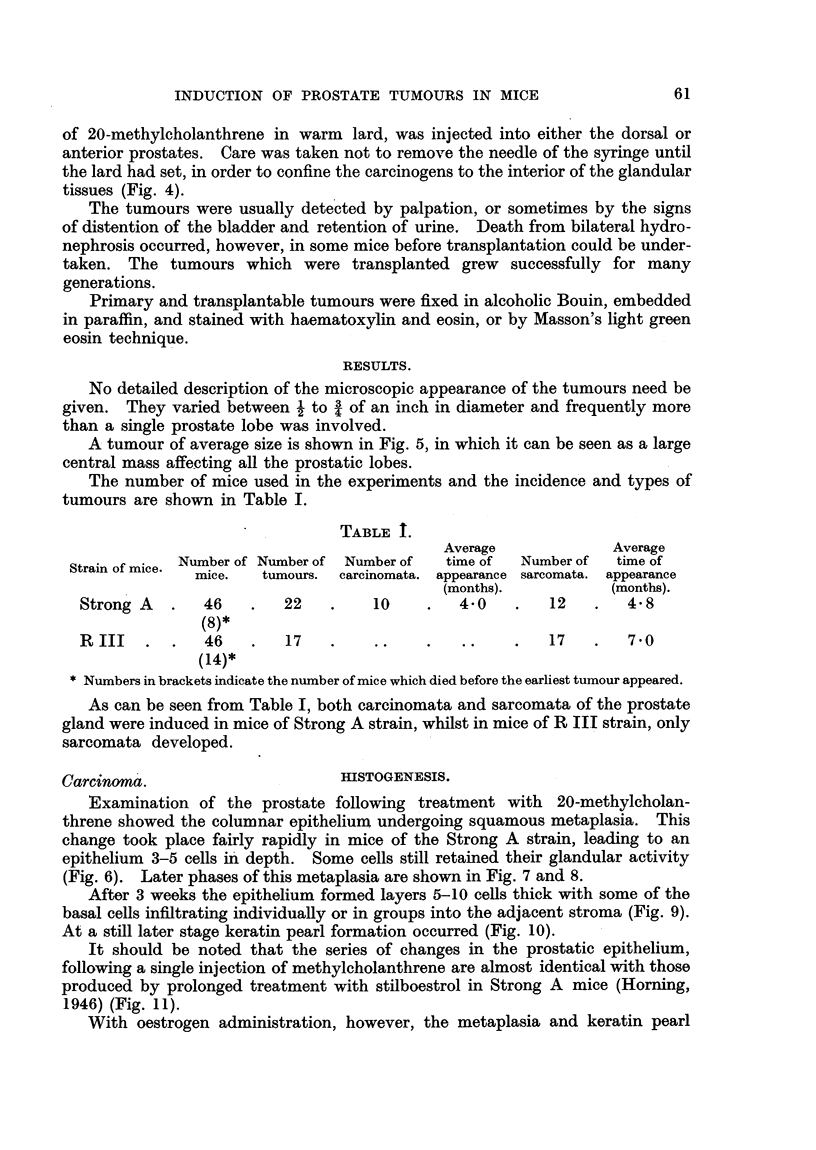

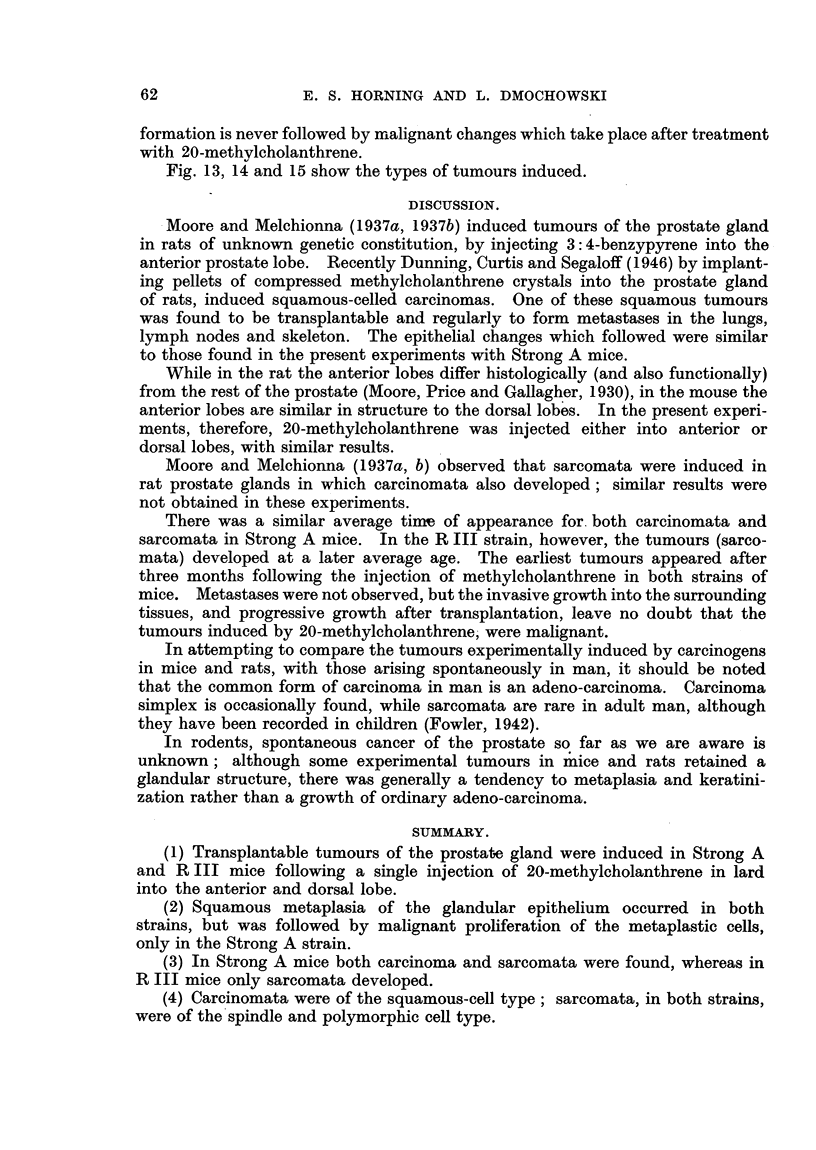

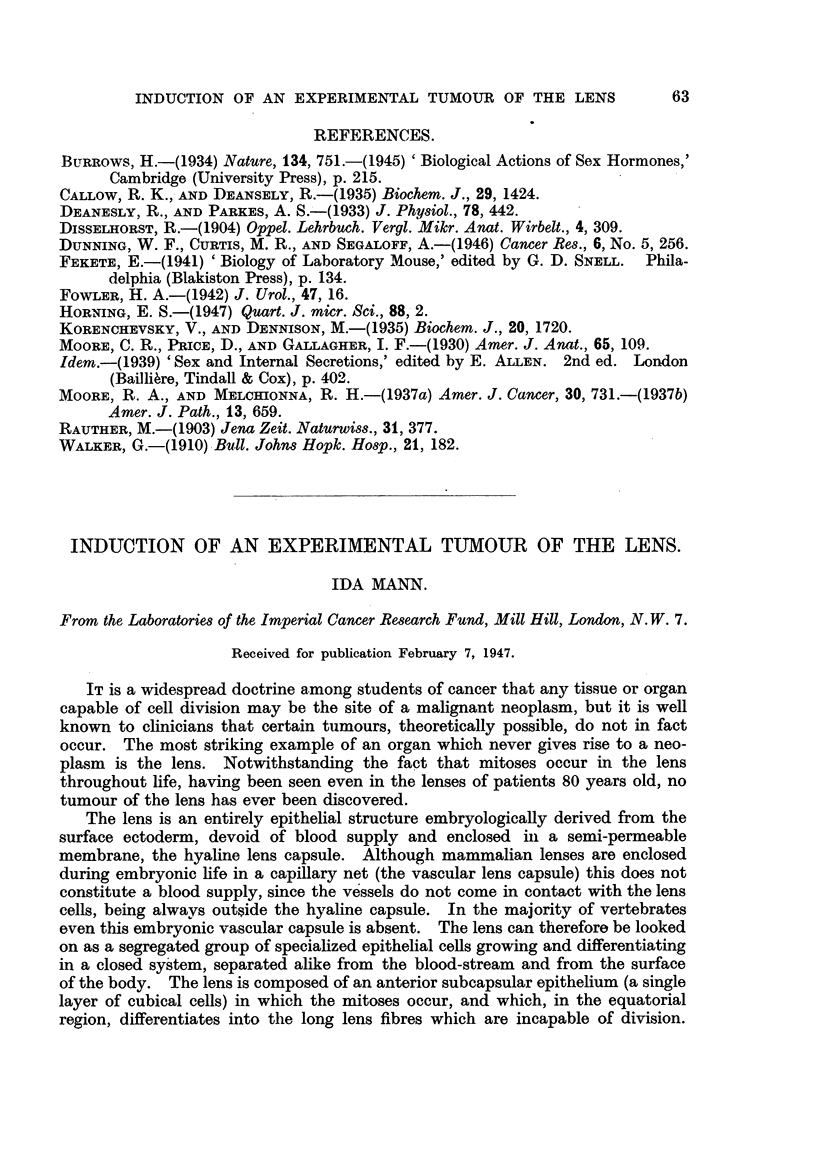


## References

[OCR_00328] Deanesly R., Parkes A. S. (1933). Size changes in the seminal vesicles of the mouse during development and after castration.. J Physiol.

[OCR_00340] Korenchevsky V., Dennison M. (1935). The assay of crystalline male sexual hormone (androsterone).. Biochem J.

